# Prevalence of asymptomatic bacteriuria among kidney transplant recipients beyond two months post-transplant: A multicenter, prospective, cross-sectional study

**DOI:** 10.1371/journal.pone.0221820

**Published:** 2019-09-06

**Authors:** Julien Coussement, Anne Scemla, Jean-Michel Hougardy, Rebecca Sberro-Soussan, Lucile Amrouche, Concetta Catalano, James R. Johnson, Daniel Abramowicz

**Affiliations:** 1 Division of Infectious Diseases, CUB-Hôpital Erasme, Université libre de Bruxelles, Brussels, Belgium; 2 Service de Néphrologie et Transplantation Adulte, Université Paris Descartes Sorbonne Paris Cité, RTRS Centaure, Labex Transplantex, Paris, France; 3 Service de Néphrologie, Dialyse et Transplantation Rénale, CUB-Hôpital Erasme, Université libre de Bruxelles, Brussels, Belgium; 4 Minneapolis Veterans Health Care System, Minneapolis, Minnesota, United States of America; 5 Department of Nephrology-Hypertension, Universitair Ziekenhuis Antwerpen and Antwerp University, Antwerp, Belgium; Medical University of Gdansk, POLAND

## Abstract

**Background:**

During routine post-kidney transplant care, most European transplant physicians screen patients for asymptomatic bacteriuria. The usefulness of this strategy is debated. To make screening cost-effective, asymptomatic bacteriuria should be prevalent enough to justify the expense, and antibiotics should improve patient outcomes significantly if asymptomatic bacteriuria is detected. Regrettably, the prevalence of asymptomatic bacteriuria among kidney transplant recipients is not well defined.

**Methods:**

To determine the prevalence of asymptomatic bacteriuria among kidney transplant recipients, we did a cross-sectional study among kidney transplant recipients undergoing routine surveillance in three outpatient transplant clinics in Belgium and France. We excluded patients who were in the first two months post-transplantation and/or had a urinary catheter. Asymptomatic participants who had a urine culture with one organism isolated at ≥ 10^5^ CFU/mL were asked to provide a confirmatory urine specimen. Asymptomatic bacteriuria was defined per Infectious Diseases Society of America guidelines.

**Results:**

We screened 500 consecutive kidney transplant recipients. Overall, the prevalence of asymptomatic bacteriuria was 3.4% (17/500 patients). It was similarly low among kidney transplant recipients who were between 2 and 12 months after transplantation (1.3%, 1/76 patients) and those who were farther after transplantation (3.8%, 16/424 patients: p = 0.49). Asymptomatic bacteriuria was significantly associated with female gender (risk ratio 3.7, 95% CI 1.3–10.3, p = 0.007) and older age (mean age: 61 ± 12 years [bacteriuric participants], versus 53 ± 15 years [non-bacteriuric participants], p = 0.03). One participant’s colistin-resistant *Escherichia coli* isolate carried the globally disseminated *mcr-1* gene.

**Conclusions:**

Among kidney transplant recipients who are beyond the second month post-transplant, the prevalence of asymptomatic bacteriuria is low. Further studies are needed to ascertain the cost-effectiveness of a screen-and-treat strategy for asymptomatic bacteriuria in this population.

## Introduction

According to a recent European survey, more than 90% of transplant physicians systematically screen for asymptomatic bacteriuria when kidney transplant recipients attend the outpatient clinic for follow-up care [1]. Most physicians perform life-long screening for bacteriuria (i.e., from transplant until death or graft loss) [1]. When a kidney transplant recipient is found to have asymptomatic bacteriuria, transplant physicians in Europe and elsewhere often administer antibiotics, either systematically or in specific circumstances (e.g., during the first months after transplant, or if the patient has a recent history of symptomatic urinary tract infection or an elevated urine leucocyte count) [1, 2]. This screen-and-treat strategy is based on the premise that detection and antimicrobial treatment of asymptomatic bacteriuria can improve patient outcomes, specifically by reducing the risk of subsequent graft pyelonephritis.

Among kidney transplant recipients, graft pyelonephritis is common and may present with few typical manifestations of kidney infection due to graft denervation and use of immunosuppressive agents [3, 4]. Although previously expert opinion and retrospective studies have supported a screen-and-treat strategy for asymptomatic bacteriuria post-kidney transplantation [5–8], recent data from prospective studies have questioned this practice. Indeed, in one such study < 10% of kidney transplant recipients with untreated asymptomatic bacteriuria developed pyelonephritis during a median follow-up of 17 months [9]. Additionally, a recent Cochrane review found antibiotics to have uncertain effects on preventing pyelonephritis and other types of urinary tract infection among kidney transplant recipients with asymptomatic bacteriuria [9–11]. This question is critically important because antibiotics use can cause definite harms, including allergic reactions, direct toxicity, drug-drug interactions, antimicrobial resistance selection, and *Clostridioides difficile-*associated diarrhea.

The effectiveness of the screen-and-treat strategy for asymptomatic bacteriuria is controversial primarily after the first two months post-kidney transplant. By contrast, during the first two months post-transplant most transplant physicians systematically treat asymptomatic bacteriuria, due to patients' typically high level of immunosuppression and routine use of urinary catheters during the immediate post-transplant period (i.e., bladder catheter, ± ureteral stent to prevent urological complications including ureterovesical anastomosis leak and ureteric stenosis) [1]. It seems unlikely that this practice will be abandoned in the near future, because kidney transplant recipients who are in the first two months post-transplantation were/are excluded from all completed and ongoing randomized controlled trials evaluating a screen-and-treat strategy for post-transplant asymptomatic bacteriuria [10].

For any screening program to be cost-effective, two criteria must be satisfied: (i) the targeted condition should be sufficiently prevalent in the screened population to justify the expense and possible hazards of screening, and (ii) an intervention should be available that significantly and cost-effectively improves patient outcomes when the condition is detected. To address the first criterion in the context of a screen-and-treat approach for post-kidney transplant asymptomatic bacteriuria, it is necessary to know how frequently asymptomatic bacteriuria is detected in the screened population, i.e., kidney transplant recipients undergoing post-transplant follow-up surveillance.

To our knowledge, the prevalence of asymptomatic bacteriuria among kidney transplant recipients remains to be precisely determined. In contrast, its cumulative incidence has been reported to be between 4 and 51%, depending on duration of follow-up and other variables [5, 9, 12–18]. However, to evaluate the cost-effectiveness of a screen-and-treat strategy, it would be more useful to assess the prevalence of asymptomatic bacteriuria rather than its cumulative incidence, which depends on how frequently post-transplantation surveillance urine cultures are done.

Additionally, several of the cited studies did not use the Infectious Diseases Society of America (IDSA) definition for asymptomatic bacteriuria, and most were retrospective–creating uncertainty regarding whether the diagnosis of asymptomatic bacteriuria was based on a urine collection technique that reduces contamination by bacteria colonizing the distal urethra and genital mucosa (e.g., midstream collection) [19]. Moreover, these studies included only patients who were in the first month(s) or year(s) post-transplantation, so likely overestimated the frequency of asymptomatic bacteriuria among long-term kidney transplant recipients [14, 20]. Finally, some were done in settings that may differ from the current European practice, e.g., by not using cotrimoxazole prophylaxis and/or removing urine catheters relatively late post-transplant.

Accordingly, we performed a cross-sectional study to determine the current prevalence of asymptomatic bacteriuria among kidney transplant recipients at our centers after the first two months post-transplant.

## Material & methods

### Study design

We performed a cross-sectional study at two transplant centers in Belgium (Hôpital Erasme, Brussels and UZ Antwerpen, Edegem) and one in France (APHP-Hôpital Necker, Paris). Our primary objective was to determine the prevalence of asymptomatic bacteriuria among kidney transplant recipients attending the outpatient clinic for routine post-transplant care. We took advantage of the fact that, at all study sites, a urine culture to screen for asymptomatic bacteriuria is done routinely at each post-transplant visit. The Erasme Hospital ethics committee provided approval before study initiation (ref: P2018/439). Need for written consent was waived by the Erasme Hospital ethics committee given the nature of the study. All data used in this study were anonymized prior to access and analysis. The reporting of this study conforms to the STROBE statement [21].

### Participants

We prospectively assessed for study participation all adult (≥ 18 years old) kidney transplant recipients who attended our outpatient clinics for routine post-transplant care. We excluded patients who were early (< 2 months) post-transplantation and/or currently had an indwelling urine catheter or performed intermittent catheterization; all other patients were included. For patients who attended the outpatient clinic more than once during the study period, only the first visit was included.

Kidney transplant recipients provided a urine specimen before their post-transplant follow-up visit, per routine clinical practice. Kidney transplant recipients whose screening urine culture yielded < 10^5^ colony-forming units (CFU)/mL were considered not to be bacteriuric, so were not asked to provide a confirmatory sample. By contrast, those whose screening culture yielded one organism(s) at ≥ 10^5^ CFU/mL were considered as possibly bacteriuric, and were asked to provide a second urine specimen within seven working days. For this confirmatory sample, participants were re-instructed by the study team regarding the preferred contamination-minimizing urine collection technique, i.e., midstream collection after cleansing of the urethral meatus.

### Setting

During the study period, the three participating centers together performed around 340 kidney transplants annually (range: 50 to 210 per center). Bladder catheters usually were removed within the first week post-transplant, and ureteral stents were removed between 10 days and 6 weeks post-transplant. Cotrimoxazole was used for from three months post-transplant to lifelong, depending on the center, to prevent *Pneumocystis jirovecii* pneumonia. At all three centers, post-transplantation patients were followed regularly at the outpatient clinic. For patients with asymptomatic bacteriuria, use of antibiotic therapy was left to the physician’s discretion. All three study centers are currently participating in a multicenter randomized controlled trial comparing antibiotics versus no therapy in kidney transplant recipients with asymptomatic bacteriuria (https://www.thelancet.com/protocol-reviews/14PRT-5447).

### Data collected

Collected data included: date of the follow-up visit, age, gender, date of transplantation, presence of diabetes requiring therapy, serum creatinine and estimated glomerular filtration rate (using CKD-EPI formula) at time of study enrolment, culture results (count, organism[s], antimicrobial susceptibility results), and urinary leucocyte counts (/mm^3^). Participants were assessed directly by the local study investigators for the presence of signs/symptoms compatible with acute cystitis (e.g., dysuria, frequency, urgency) or pyelonephritis (e.g., fever, kidney pain), using pre-determined definitions. Participants whose initial urine culture yielded ≥ 10^5^ CFU/mL were queried regarding their adherence to the recommended sample collection method when obtaining their initial urine sample (midstream collection; cleaning of the urethral meatus) and were asked to submit a second (confirmatory) urine sample.

### Definitions

Per the IDSA 2005 guidelines [22], asymptomatic bacteriuria was defined–in women–as two consecutive urine specimens yielding the same organism at ≥ 10^5^ CFU/mL, from a patient without signs or symptoms suggesting urinary tract infection. In men, a single specimen sufficed. Pyuria was defined as > 25 leukocytes/mm^3^ in urine.

### Microbiological considerations

Urine testing was performed locally. An automated analyzer (either SediMAX [Menarini] or UF-5000 [Sysmex], depending on study site) was used to quantify urine leukocytes. Urine cultures were performed using either cysteine-lactose-electrolyte-deficient agar (bioMérieux) or UriSelect 4 agar (BioRad), depending on study site. Matrix-assisted laser desorption/ionization time-of-flight mass spectrometry was used for microbial identification. Asymptomatic bacteriuria isolates underwent antimicrobial susceptibility testing using either the VITEK-2 system (bioMérieux) or the disk diffusion method and current European Committee on Antimicrobial Susceptibility Testing breakpoints. Colistin resistance (if suspected) was confirmed using broth microdilution.

### Study size

A pre-study sample size calculation was done to estimate the needed number of participants. Based on related data from non-transplant studies [22], we projected an 8% prevalence of asymptomatic bacteriuria among adult kidney transplant recipients attending the outpatient clinic. Assuming a precision of 2.5% and a type 1 error rate of 5%, a sample size of 453 participants was determined using OpenEpi (http://www.openepi.com/). We therefore decided to include a total of 500 participants.

### Data statistical analysis

After all data were verified, statistical analysis was performed using STATA 15. No data were missing. We opted to present categorical variables as numbers and frequencies, and continuous variables as means (± standard deviation) or medians (interquartile range), as appropriate. To estimate the prevalence of asymptomatic bacteriuria, the Wilson method was used to compute the confidence interval (CI) of the proportion. Categorical variables were compared using a chi-square test or Fisher’s exact test, as appropriate. Normally distributed continuous variables were compared using a t-test. Non-normally distributed continuous variables were compared using the Mann-Whitney test. A two-tailed p-value < 0.05 was considered statistically significant.

## Results

### Participants

**Fig 1:**
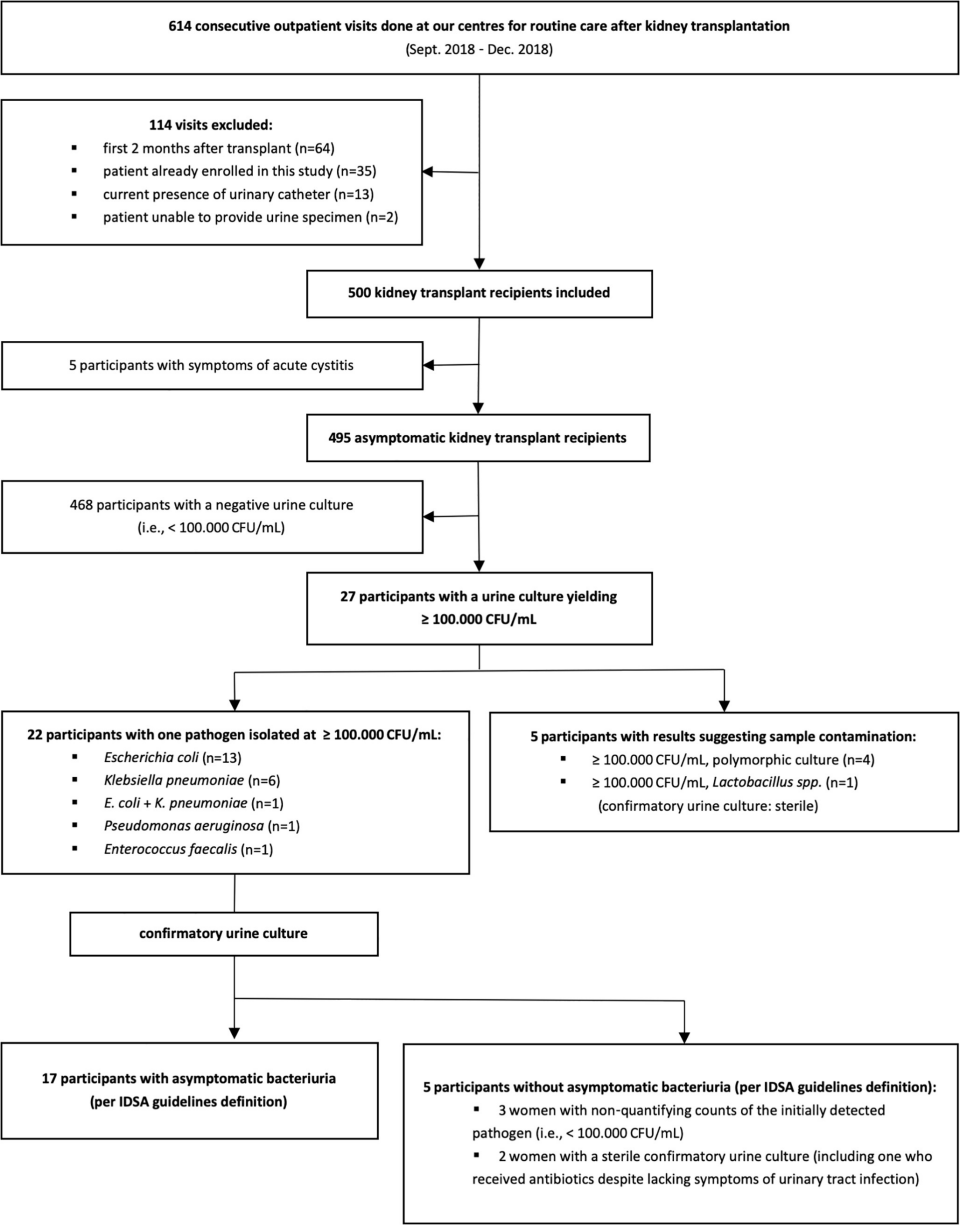
Flow chart.

We screened 579 unique patients during 614 consecutive outpatient visits at our centers for routine care after kidney transplantation. We enrolled the 500 unique participants who met all inclusion criteria (303 men and 197 women), and excluded 114 visits (35 because the patient had already been enrolled at an earlier visit, 79 due to various other exclusionary factors, Fig 1). Characteristics of the 500 unique participants are described in Table 1.

**Table 1:** Characteristics of study participants, and parameters associated with asymptomatic bacteriuria.

	Participants without asymptomatic bacteriuria (n = 483)	Participants with asymptomatic bacteriuria (n = 17) *	p
**Female gender, n (%)**	185 (38%)	12 (71%)	0.007
**Age (years), mean ± SD**	53 ± 15	61 ± 12	0.03
**Time from transplant (months), median (IQR)**	61 (23–137)	61 (45–162)	0.3
**Uropathy as the cause of end-stage kidney disease** **, n (%)	47 (10%)	1 (6%)	0.6
**Treated diabetes, n (%)**	135 (28%)	4 (24%)	0.8
**eGFR (ml/min/1.73 m**^**2**^**), mean ± SD**	54 ± 21	45 ± 16	0.09
**Use of cotrimoxazole prophylaxis at time of urine sample collection, n (%)**	186 (39%)	9 (53%)	0.2
**Pyuria (i.e., > 25 leucocytes/mm**^**3**^ **in urine), n (%)**	56 (12%)	15 (88%)	< 0.001

* Asymptomatic bacteriuria was defined using the Infectious Diseases Society of America 2005 definition

** None of the subjects included in this study had a history of bladder augmentation

eGFR: estimated glomerular filtration rate (evaluated using CKD-EPI formula); IQR: interquartile range; RR (95% CI): risk ratio with 95% confidence interval; SD: standard deviation

### Prevalence of asymptomatic bacteriuria

Among the 500 kidney transplant recipient participants, 22 (4.4%) were asymptomatic and had a first urine specimen that yielded one bacterial pathogen isolated at ≥ 10^5^ CFU/mL (Fig 1). Nearly all of these 22 participants declared having collected the urine sample appropriately (midstream collection in 21/22 [95%]; cleaning of the urethral meatus in 19/22 [86%]). All 22 positive urine cultures triggered the confirmatory culture of a second urine specimen, which confirmed the presence of asymptomatic bacteriuria (per IDSA guidelines definition) in 17/22 participants, for an overall asymptomatic bacteriuria prevalence of 3.4% (17/500: 95% CI 2.1 to 5.4%). The remaining 5/22 participants were asymptomatic women whose confirmatory urine cultures yielded either non-qualifying counts (i.e., < 10^5^ CFU/mL) of the initially detected pathogen (n = 3) or no growth (n = 2); one of the latter participants had received antibiotics from her provider despite lacking symptoms of urinary tract infection.

The prevalence of asymptomatic bacteriuria was significantly higher among female participants (12/197, 6.1%) than among male participants (5/303, 1.7%, p = 0.007). The prevalence of asymptomatic bacteriuria was not significantly different between kidney transplant recipients who were between 2 and 12 months after transplantation (1.3%, 1/76 patients) and those who were farther after transplantation (3.8%, 16/424 patients: p = 0.49).

### Asymptomatic bacteriuria organisms and resistance profiles

The organisms causing these 17 episodes of asymptomatic bacteriuria included *Escherichia coli* (n = 10, 59%), *Klebsiella pneumoniae* (n = 5, 29%), *Pseudomonas aeruginosa* (n = 1, 6%), and *Enterococcus faecalis* (n = 1, 6%). Among the 15 Enterobacteriaceae (i.e., *E*. *coli* and *K*. *pneumoniae*) isolates, resistance was documented to ciprofloxacin in 2 (13%), extended-spectrum cephalosporins in 3 (20%), cotrimoxazole in 9 (60%), and amoxicillin in 11 (65%). Additionally, in one participant asymptomatic bacteriuria was due to a colistin-resistant *E*. *coli* strain (MIC 16 μg/mL) that by PCR-based detection carried the globally disseminated *mcr-1* gene

### Epidemiological correlates of asymptomatic bacteriuria

As compared with other participants, those with asymptomatic bacteriuria more commonly were female (12/17 [71%], versus 185/483 [38%], risk ratio [RR] 3.7, 95% CI 1.3 to 10.3: p = 0.007) and had pyuria (15/17 [88%], versus 56/483 [12%]: p < 0.001). They also were older (mean age: 61 ± 12 years, versus 53 ± 15 years: p = 0.03). Consequently, the prevalence of asymptomatic bacteriuria was significantly higher among female participants > 50 years old (10/122, 8.2%) than among male participants ≤ 50 years old (0/125, 0%, p = 0.001). No other study variable was associated with asymptomatic bacteriuria (Table 1).

## Discussion

This study, which to our knowledge provides the first prospective point-prevalence assessment for asymptomatic bacteriuria among kidney transplant recipients, identified asymptomatic bacteriuria in only 3.4% of kidney transplant recipients attending the outpatient transplant clinic after their second month post-transplantation. This value was much lower than anticipated. Consequently, and because in a prospective study < 10% of kidney transplant recipients with untreated asymptomatic bacteriuria developed pyelonephritis during a median follow-up period of 17 months [9], the value of a routine screen-and-treat strategy for asymptomatic bacteriuria beyond the second month post-transplantation–as is done by most European transplant physicians–is unclear.

Our cross-sectional study aimed at determining the point prevalence of asymptomatic bacteriuria among kidney transplant recipients who are after their second month post-transplantation (i.e., the proportion of kidney transplant recipients who have asymptomatic bacteriuria, at a particular point of time), which is important for evaluating the cost-effectiveness of a screen-and-treat strategy. In contrast, previous studies focused on the cumulative incidence of asymptomatic bacteriuria (i.e., the proportion of kidney transplant recipients who have at least one episode of asymptomatic bacteriuria, over a particular time period during which urine is generally tested multiple times). The cumulative incidence of asymptomatic bacteriuria has been reported to be between 4 and 51%, depending on duration of follow-up and other variables (e.g., frequency of urine testing during the follow-up period) [5, 9, 12–18]. To our knowledge, only one prospective study used the IDSA guidelines definition of asymptomatic bacteriuria to determine its cumulative incidence after kidney transplantation [9]. In this study, 39% of kidney transplant recipients had at least one episode of asymptomatic bacteriuria between 2 months and 2 years post-transplantation (frequency of screening: at least bi-monthly during the first 3 months post-transplant, monthly between 3 and 12 months, and every 1–3 months thereafter) [9].

Although post-transplant symptomatic urinary tract infections such as pyelonephritis are costly, the strategy of screening for and treating asymptomatic bacteriuria in kidney transplant recipients also has costs, and has not been shown to prevent symptomatic urinary tract infections [10]. Not screening for asymptomatic bacteriuria in kidney transplant recipients after the second month post-transplantation may reduce both direct costs (i.e., from avoidance of urine cultures and antibiotic use) and, possibly, indirect costs related to harms from antibiotics use (e.g., *Clostridoides difficile-*associated diarrhea, adverse drug effects, and antimicrobial resistance).

Antimicrobial resistance is an especially concerning threat to kidney transplant recipients. After the dissemination in the 1990s of organisms that produce extended-spectrum ß-lactamases (bacterial enzymes that confer resistance to extended-spectrum cephalosporins), and the subsequent dissemination of carbapenem-resistant organisms and the responsible genetic elements, we now face a similar process with organisms resistant to “last resort” antibiotics such as colistin [23]. This is illustrated by our detection in one study participant of asymptomatic bacteriuria due to a colistin-resistant *E*. *coli* strain carrying the globally disseminated *mcr-1* gene. The plasmid-borne *mcr-1* gene was described first in 2016, in a study from China [23]. Following that initial report, colistin-resistant *E*. *coli* isolates carrying *mcr-1* were found on all continents. This gene's dissemination may threaten our future ability to treat extensively multidrug-resistant infections. Asymptomatic carriage of plasmid-mediated colistin resistance by an outpatient in our center’s post-transplantation clinic raises concerns about possible future patient-to-patient transmission of this genetic element and a corresponding need for special preventive measures in the outpatient setting [24].

Our study has notable limitations. First, the lower-than-expected prevalence of asymptomatic bacteriuria limited power for identifying predictive factors. However, we found two demographic factors to be significantly associated with asymptomatic bacteriuria, i.e., female gender and older age. If ongoing randomized controlled trials show effectiveness for antibiotic therapy of asymptomatic bacteriuria in kidney transplant recipients [10], these two variables could be used to identify patients who potentially are most likely to benefit from a screen-and-treat strategy. Second, generalizability may be limited by our exclusion of patients who were in the first two months after kidney transplantation and/or had a urinary catheter, who may have a higher prevalence of asymptomatic bacteriuria. Finally, the fact that almost 40% of the subjects included in this study were receiving cotrimoxazole prophylaxis at time of urine sample collection may have reduced the prevalence of asymptomatic bacteriuria.

Our study also has notable strengths. First, systematic screening for asymptomatic bacteriuria at multiple centers in consecutive outpatients presenting for routine post-transplantation care likely provided a sample representative of the population of interest (i.e., non-catheterized kidney transplant recipients who are beyond the second month post-transplantation). Second, conformity with the IDSA guidelines definition of asymptomatic bacteriuria limited the risk of misclassifying contaminated urine samples as episodes of asymptomatic bacteriuria. Third, the prospective design allowed us to ensure that urine specimens were collected using a contamination-minimizing technique, i.e., midstream collection after cleansing of the urethral meatus.

In conclusion, the point prevalence of asymptomatic bacteriuria was only 3.4% among kidney transplant recipients beyond the second month post-transplantation, according to cross-sectional screening with rigorous confirmation. This low prevalence, together with recent evidence that antibiotic therapy for post-transplantation asymptomatic bacteriuria may fail to improve patient outcomes, calls into question the usefulness of a screen-and-treat strategy for asymptomatic bacteriuria among kidney transplant recipients after the second month post-transplantation. Ongoing randomized controlled trials should further clarify the value of a screen-and-treat strategy for post-transplantation asymptomatic bacteriuria [10].

## Supporting information

S1 Table Dataset. (XLSX)

## Abbreviations

CFU: colony-forming units
CI: confidence interval
IDSA: Infectious Diseases Society of America
RR: risk ratio
